# The effect of weekly interactive text-messaging on early infant HIV testing in Kenya: a randomised controlled trial (WelTel PMTCT)

**DOI:** 10.1038/s41598-021-00972-6

**Published:** 2021-11-22

**Authors:** Björn Nordberg, Winfred Mwangi, Mia Liisa van der Kop, Edwin Were, Eunice Kaguiri, Anna E. Kågesten, Erin E. Gabriel, Richard T. Lester, Jonathan Mwangi, Anna Mia Ekström, Susanne Rautiainen

**Affiliations:** 1 Department of Global Public Health, Karolinska Institutet, Stockholm, Sweden; 2 Department of Infectious Diseases, Helsingborg Hospital, Helsingborg, Sweden; 3 Directorate of Reproductive Health, Moi Teaching and Referral Hospital, Eldoret, Kenya; 4 Department of Reproductive Health, Moi University, Eldoret, Kenya; 5 Partners in Prevention, Moi University, Eldoret, Kenya; 6 Department of Medical Epidemiology and Biostatistics, Karolinska Institutet, Stockholm, Sweden; 7 Department of Medicine, Division of Infectious Diseases, University of British Columbia, Vancouver, Canada; 8 Department of Infectious Diseases, South Central Hospital, Stockholm, Sweden; 9 Astrid Lindgren’s Children’s Hospital, Karolinska University Hospital, Stockholm, Sweden; 10 Division of Preventive Medicine, Brigham and Women’s Hospital, Boston, USA

**Keywords:** Public health, HIV infections

## Abstract

Mother-to-child transmission of HIV remains a significant concern in Africa despite earlier progress. Early infant diagnosis (EID) of HIV is crucial to reduce mortality among infected infants through early treatment initiation. However, a large proportion of HIV-exposed infants are still not tested in Kenya. Our objective was to investigate whether weekly interactive text-messages improved prevention of mother-to-child transmission (PMTCT) of HIV care outcomes including EID HIV testing. This multicentre, parallel-group, randomised, open-label trial included six antenatal care clinics across western Kenya. Pregnant women living with HIV, aged 18 years or older, with mobile phone access, were randomised in a 1:1 ratio to weekly text messages that continued until 24 months postpartum, asking “How are you?” (“Mambo?”) to which they were asked to respond within 48 h, or a control group. Healthcare workers contacted participants reporting problems and non-responders by phone. Participants in both groups received routine PMTCT care. The prespecified secondary outcome reported in this paper is EID HIV testing by eight weeks of age (blinded outcome assessment). Final 24-months trial results will be published separately. We estimated risk ratios using Poisson regression with robust standard errors. Between June 2015–July 2016, we screened 735 pregnant women, of whom 600 were enrolled: 299 were allocated to the intervention and 301 to the control group. By eight weeks of age, the uptake of EID HIV testing out of recorded live births was 85.5% in the intervention and 84.7% in the control group (71.2% vs. 71.8% of participants randomised, including miscarriages, stillbirths, etc.). The intention-to-treat risk ratio was 0.99; 95% CI: 0.90–1.10; *p* = 0.89. The proportion of infants diagnosed with HIV was 0.8% in the intervention and 1.2% in the control group. No adverse events were reported. We found no evidence to support that the WelTel intervention improved EID HIV testing. A higher uptake of EID testing than expected in both groups may be a result of lower barriers to EID testing and improved PMTCT care in western Kenya, including the broader standard use of mobile phone communication between healthcare workers and patients. (ISRCTN No. 98818734. Funded by the European-Developing Countries Clinical Trial Partnership and others).

## Introduction

Mother-to-child transmission (MTCT) of HIV remains a significant concern in many African countries^1^. Estimates indicate that of the 150 000 children aged 0–14 years that were infected with HIV worldwide in 2019, the majority occurred in sub-Saharan Africa (SSA)^2^. Recent Joint United Nations Programme on HIV/AIDS (UNAIDS)’ data show disruptions in prevention of mother-to-child transmission (PMTCT) services in a majority of low-and middle income countries as a consequence of reduced access to basic healthcare during the COVID-19 crisis^3^. Recent modelling suggests that this may lead to a 40–80% increase of new paediatric HIV infections in high-burden countries in SSA^4^. To reduce infant mortality, the World Health Organization (WHO) strongly recommends early infant diagnostic (EID) HIV testing around six weeks of age for all HIV-exposed infants, and the rapid initiation of life-long antiretroviral therapy (ART) among infants diagnosed with HIV^5^. Despite this recommendation, only 68% of HIV-exposed infants were tested for HIV by eight weeks of age in eastern and southern Africa in 2019, before the start of the COVID-19 pandemic^2^. Kenya had the sixth highest number of vertically transmitted HIV cases globally, with an estimated 6800 infants newly infected in 2019 (5% of new HIV cases in infants globally)^1^. In 2015, at the start of this trial, the Kenyan national EID HIV testing coverage was only 54%^6^.

Most countries, including Kenya, have implemented the WHO PMTCT Option B + guidelines, in which all pregnant women living with HIV receive life-long ART^7^. This has increased access to ART for pregnant women living with HIV in Kenya and other high-burden SSA countries^1^. However, concerns about involuntary HIV disclosure and HIV-related stigma^8–12^, a recent HIV diagnosis^13^, long distance and long time to reach the clinic^14^, transportation costs^15^, and negative healthcare staff treatment^16^ are barriers to pregnant women’s participation in PMTCT care. Therefore, there is a need for interventions that improve the uptake of PMTCT services without jeopardzing pregnant women’s privacy and confidentiality, including EID HIV testing^17^.

Using text-messaging to improve communication between healthcare workers and patients is a promising concept given the rapid increase in mobile phone use in SSA, including Kenya^18^. Previous randomised controlled trials (RCT) of mobile text message interventions have shown mixed results of these interverventions on the uptake of EID HIV testing^19–22^. Before the implementation of WHO’s PMTCT Option B+ guidelines, two African RCTs reported that mobile text messages significantly improved EID HIV testing, one of 388 pregnant women in Kenya 2012–2013^19^ and one RCT of 522 pregnant women in Mozambique 2011–2012^20^. In contrast, two cluster RCTs of 2472^21^ and 550 pregnant women in Kenya^22^, as well as a retrospective study of 235 pregnant women in South Africa^23^, reported no significant effects of text-messaging interventions on EID HIV testing rates.

The WelTel intervention is an interactive text-messaging check-in service in which participants are sent automatically-generated, anonymous, weekly text messages asking “How are you?”^24–26^. Participants are instructed to respond within 48 h either that they are well or that they have an issue. If they report a problem or do not respond within the requested timeframe, they are contacted by healthcare workers from their local antenatal care (ANC) clinic. The overall aim of the WelTel PMTCT RCT was to support retention in both ANC and post-natal care up to 24 months for women living with HIV. Compared to previously tested mobile text-messaging interventions on EID HIV testing^19–22^, the WelTel intervention may have an advantage of early identification of problems among patients, which allows healthcare workers to provide early support, counselling and medical assistance. Qualitative studies have suggested that the WelTel intervention increases patient involvement^27^ and improves communication between patients and healthcare workers^28^. The WelTel intervention demonstrated improvements in adherence to ART and HIV viral load suppression in an RCT of 538 non-pregnant people living with HIV in Kenya^25^. In contrast, another RCT of 700 non-pregnant people living with HIV in Kenya reported improved self-perceived quality of life, but no significant effect on retention during first year of HIV care^26^.

No previous study has investigated the effect of the WelTel intervention on retention in care among pregnant and postpartum women living with HIV, enrolled in a PMTCT program, including their new-born infants. It is particularly important to keep pregnant and postpartum women living with HIV and their new-born infants retained in care to decrease the risk of MTCT and to enable EID of HIV and ART initiation. This paper reports the effect of a weekly interactive text message intervention (WelTel) delivered to women enrolled in PMTCT Option B+ care in western Kenya on the proportion of EID HIV testing among HIV-exposed infants by eight weeks of age. EID HIV testing was a prespecified secondary outcome that occurred early during follow-up of the WelTel PMTCT trial. The results are published on their own for proper comparison to previous studies of text-message interventions in PMTCT care, that mainly has focused on EID HIV testing. The primary trial outcome (retention in PMTCT care up to 24 months postpartum) will be published separately^29^.

## Methods

### Study design and setting

**Figure 1:**
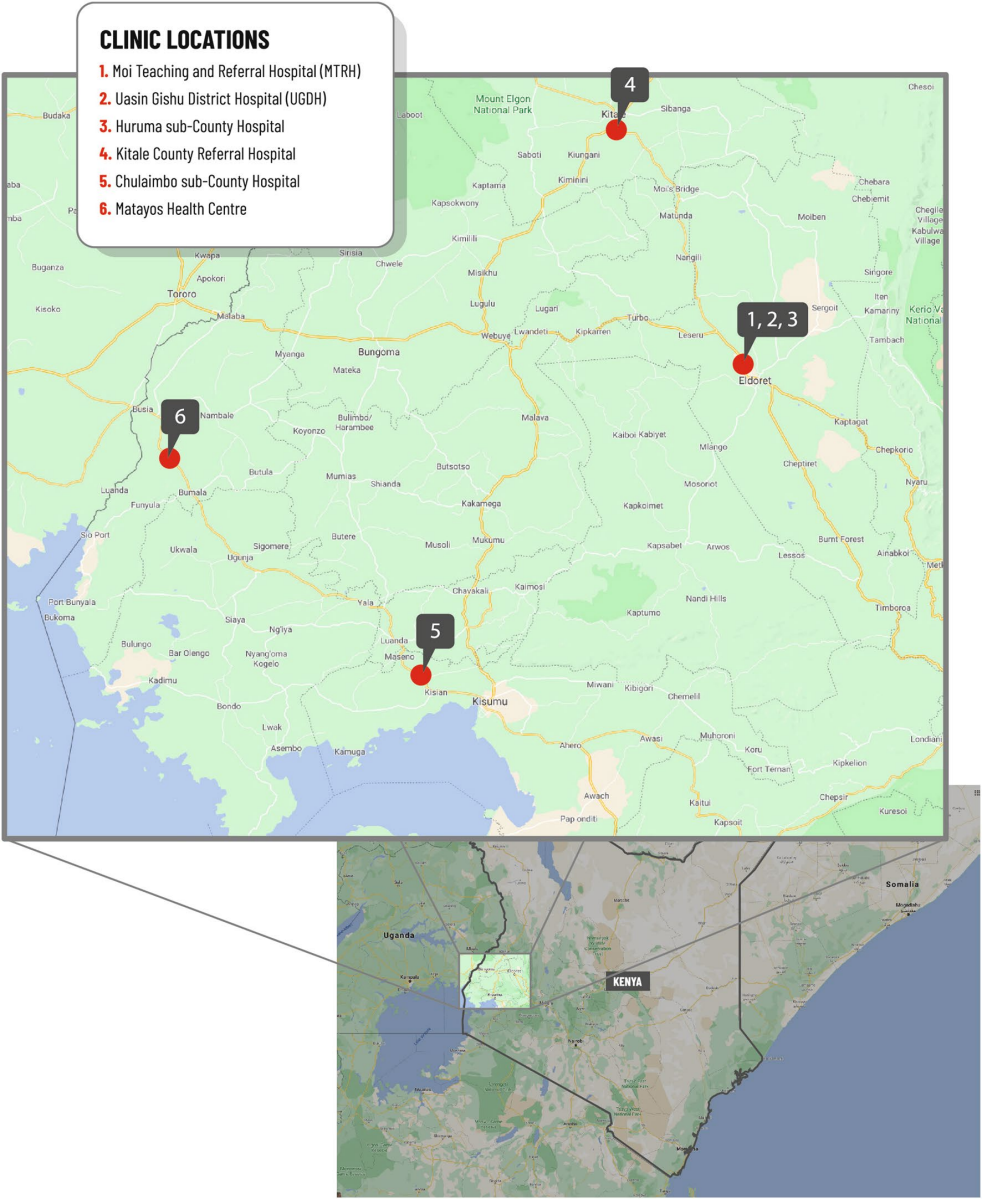
Location of study sites in western Kenya.

WelTel PMTCT was a multicentre parallel-group randomised controlled open-label trial carried out at six ANC clinics in western Kenya. The primary trial endpoint was retention in PMTCT care up to 24 months postpartum among women living with HIV and their HIV-exposed infants. This paper is the first trial report on a prespecified secondary outcome to investigate the first step in the postpartum PMTCT cascade, EID HIV testing by eight weeks of age^29^. The ANC clinics in the study are government-run and part of the Academic Model Providing Access to Healthcare (AMPATH) program. Clinics included Moi Teaching and Referral Hospital (MTRH), Uasin Gishu District Hospital (UGDH), Huruma sub-County Hospital, Kitale County Referral Hospital, Chulaimbo sub-County Hospital, and the Matayos Health Centre (Fig. 1). MTRH, UGDH and Huruma sub-County Hospital are located in Eldoret, the fifth most populated town in Kenya. The clinics in Kitale, Chulaimbo and Matayos are located in different parts of western Kenya. Overall, the ANC clinics involved in the trial were spread over a large geographical area and serve urban as well as rural populations. All participants provided informed consent and ethics approval was obtained from the Institutional Research and Ethics Committee at Moi University, Kenya (FAN: IREC 1292) and the Regional Ethics Committee, Stockholm, Sweden (2018/742-31/1). All trial procedures were performed in accordance with relevant guidelines and regulations. The study was registered at the international standard randomised controlled trial number (ISRCTN) registry (ISRCTN98818734, registration date: 09/12/2014) and the trial protocol has been published elsewhere^24^.

### Participants

**Figure 2:**
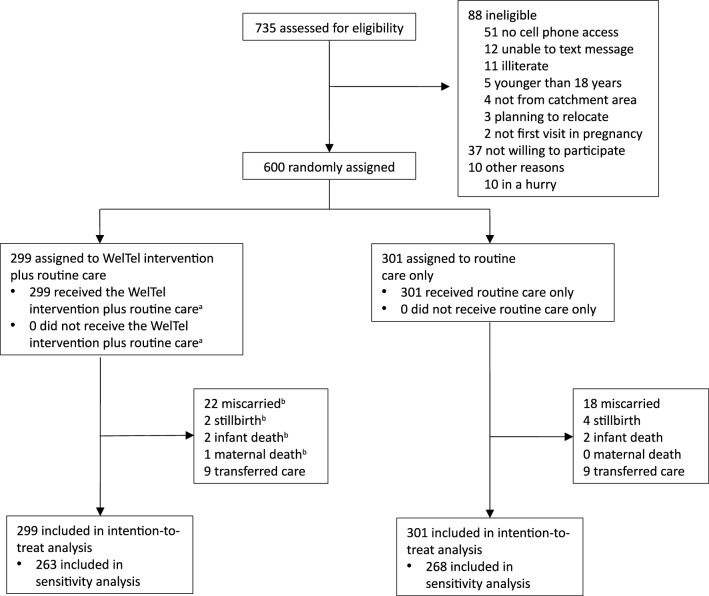
Flow chart of screening, enrolment, reasons for non-participation and randomisation of participants. ^a^One participant withdrew from receiving text messages before the infant was born, after having received 31 messages. The participant agreed to continued follow-up and was included in intention-to-treat analysis as a participant of the intervention group. ^b^WelTel intervention was stopped in case of miscarriage, stillbirth, infant death, or maternal death.

Between June 25th, 2015 and July 5th, 2016, pregnant women living with HIV aged ≥ 18 years and presenting to their first ANC visit in their current pregnancy were invited to participate in the trial. HIV diagnosis was based on two repeated Determine™ or Colloidal Gold blood tests, or referral from a HIV comprehensive care clinic for those with known HIV infection. To be eligible, women had to have access to a mobile phone, be able to respond or have someone in close contact who could respond to the text messages, and be a resident of the ANC clinic catchment area. Women who planned to relocate from the ANC clinic catchment area, and who were not willing to be followed up until 24 months postpartum were excluded. Infants born to trial participants were also included in follow-up (Fig. 2).

### Randomisation and blinding

Participants were interviewed face-to-face by a trained research assistant in Kiswahili or English upon trial enrolment. Participants were thereafter randomly allocated by the research assistant to the intervention or control group using a 1:1 allocation ratio. Randomisation was performed at each ANC clinic separately using individual, opaque, sealed envelopes. To ensure balance of participants’ baseline characteristics between the two study groups, a computer-generated non-disclosed permuted-block randomisation scheme with block sizes equal to four was generated by an independent statistician at the Karolinska Institute, Stockholm, Sweden. The randomisation scheme was generated for each clinic separately. The randomisation sequence was created using the ralloc command in Stata (StataCorp. 2015. *Stata Statistical Software: Release 14*. College Station, TX: StataCorp LP). The trial was open-label and unmasked as the intervention required overt participation. The trial was however assessor-blinded to laboratory staff that analysed the EID HIV tests and to the authors of the paper. The randomisation code was disclosed to the research team only after the trial had ended and data management was complete in September 2020.

### The WelTel intervention

Every Monday morning, a mobile text message in Kiswahili was automatically sent asking “How are you?” (“Mambo?” in Kiswahili) from a digital platform using a generic program number saved to the participant’s phone under a name of their chosing. The participants were instructed to respond within 48 h that they were either doing well (e.g. “Sawa” in Kiswahili) or that they had a problem (e.g. “Shida” in Kiswahili). The participants’ responses (in Kiswahili or English), and instances of non-response were automatically registered in the platform. Reported problems were automatically forwarded to a mobile phone at the participant’s local ANC clinic where they were followed up by a healthcare worker within 48 h. A list of non-responses was made every week by a study coordinator and delivered (by paper or text message) to the local ANC clinic by a research assistant, where they were followed up by a healthcare worker. Healthcare workers contacted the participants by telephone to determine the nature of the problem or reason for not responding and then provided assistance. Outcomes of the follow-up calls were recorded in a log by the healthcare worker. The WelTel intervention continued until 24 months postpartum, except in case of a miscarriage, stillbirth, infant death, maternal death, or participant withdrawal from the intervention, when text messages were discontinued. Apart from weekly text-messaging, participants in the intervention group received the same PMTCT care as the control group. Intervention group participants were informed that the messaging did not replace routine clinic services, that scheduled appointments should be honoured, and that all emergencies should be handled by usual means.

### Control group

Participants in the control group received routine PMTCT care, based on the WHO’s PMTCT Option B+ guidelines^7^, which included a defaulter tracing outreach program that was initiated by AMPATH in western Kenya in 2005 (i.e. patients who missed one or more scheduled appointments were traced, first by telephone, then by household visit)^30,31^.

### Outcomes

EID HIV testing was a secondary trial outcome of the WelTel PMTCT trial^24^. It was defined as the proportion of HIV-exposed infants who had an early HIV test by eight weeks of age. The definition of an EID HIV test was modified after the study protocol was published but before the statistical analyses were undertaken^29^. The definition of an EID test in the published study protocol was ‘HIV-exposed infants tested for HIV within eight weeks of birth, measured as HIV-exposed infants with known HIV status at age 10 weeks’. The change was made to be consistent with UNAIDS’ most recent definition of an EID HIV test (i.e. the percentage of infants born to women living with HIV receiving a virological test for HIV within two months of birth)^32^. During participant follow-up, ANC clinics involved in the trial piloted at-birth HIV testing. All infant HIV tests prior to eight weeks of age (including at-birth tests), were included to define EID HIV testing by eight weeks of age. In case of an at-birth test, the participants were instructed to have a second infant HIV test at around six weeks of age. To exclusively assess EID HIV testing around six weeks of age, we also analysed the proportion of infants tested for HIV between four to eight weeks of age. In addition, we calculated the proportion of infants diagnosed with HIV based on EID HIV testing. Information about EID testing was extracted from patient files, patient registers and the Kenyan National AIDS & STI Control Program (NASCOP) database.

### Other measures

Information on socio-demographic and HIV-related characteristics was collected through interviews at trial enrolment, including: age (18–24, 25–29, 30–34, 35–44 years); education (≤ primary schooling, secondary schooling, higher education); married or living with a partner (yes, no); employment status (working outside the household i.e. employed, self-employed, casual labour, farm work, or not working outside the household i.e. unemployed, homemaker, student); time since HIV diagnosis (< 6 months, ≥ 6 months); travel time to clinic (< 1 h, ≥ 1 h); HIV status disclosure to someone (yes/no); and HIV disclosure to a partner (yes/no). HIV status disclosure to a partner excluded participants that were not married or living with a partner. Overall, there were few participants from the Matayos clinic. Therefore, based on similarities between the Chulaimbo and Matayos clinics, participants from these clinics were combined in regression models and subgroup analyses.

### Statistical analysis

The sample size of the WelTel PMTCT trial was based on trial’s primary endpoint^24^. A sample size of approximately 300 in each study group was estimated to have at least 80% power to detect an 11% difference in the primary endpoint using α = 0.05. We expected 30% of participants in the control group to be retained at 24 months. The primary analysis of the effect of the intervention on EID HIV testing was intention to treat, i.e. all women were analysed according to the treatment group to which they were originally allocated. We used descriptive statistics to summarize demographic statistics of the study population. For the outcome of EID HIV testing by eight weeks and for the analysis of infants tested between four to eight weeks of age, we used Poisson regression with robust standard errors to estimate risk ratios (RR), rather than odds ratios as infant testing is not a rare event. Poisson regression with robust standard errors is known to perform well over a wide range of settings, and could thus be pre-specified with confidence in the trial’s statistical analysis plan published prior to data analyses^29,33,34^. We ran each of these models adjusting for women’s age group at baseline, time from HIV diagnosis to trial enrolment, and ANC clinic of enrolment.

We also ran these analyses excluding mother-infant pairs with a record of miscarriage, stillbirth, infant death, or maternal death prior to eight weeks of age, and a record of transfer of care before the infant was eight weeks old, which included recorded transfers of the mother prior to giving birth. We conducted sensitivity analyses to determine whether there was an association between these exclusions and trial group allocation using logistic regression. Exploratory hypothesis-generating subgroup analyses were performed using Poisson regression with robust standard errors. All p-values are unadjusted, and all models are reported. Analyses were performed using StataCorp. 2017. *Stata Statistical Software: Release 15*. College Station, TX: StataCorp LLC.

## Results

Between June 25th, 2015, and July 5th, 2016, 735 pregnant women were screened, 600 of whom were enrolled (Fig. 2): 299 were randomly assigned to the WelTel intervention and 301 to the control group (routine care). Not having access to a mobile phone was the most common reason for non-inclusion. One participant withdrew from receiving text messages before the infant was born, after having received 31 messages, but agreed to be followed-up and was therefore included in intention-to-treat analyses in the intervention group. There were no other participant withdrawals from trial before their infants were eight weeks old. All participants in the intervention group received the study intervention. To our knowledge, no participant in the control group received the intervention. Follow-up of of the EID HIV testing outcome concluded on March 23rd, 2017, when the last infant reached eight weeks of age. Follow-up of the WelTel PMTCT trial cohort concluded on July 26th, 2019.

Baseline characteristics of the participants are presented in Table 1. More than half (54.0%) had secondary schooling or higher education. Most of the trial participants were married or living with a partner (81.3%), not working outside the household (61.5%) and diagnosed with HIV ≥ 6 months before trial enrolment (70.5%). A majority of the participants who were married or living with a partner had disclosed their HIV status to their partner (71.1%). Overall, baseline characteristics were balanced between the intervention and control groups, with a few more women working outside the household and with ≤ primary schooling as highest education in the intervention group.

**Table 1:** Study population baseline characteristics (N = 600).

Characteristic	WelTel intervention (n = 299) n (%)	Routine care (control group, n = 301) n (%)
**Age (years)**		
18–24	67 (22.4)	69 (22.9)
25–29	79 (26.4)	89 (29.6)
30–34	83 (27.8)	83 (27.6)
35–44	70 (23.4)	60 (19.9)
	mean = 29.6	mean = 29.1
	SD = 5.9	SD = 5.5
**Highest education**		
≤ Primary schooling	147 (49.2)	129 (42.9)
Secondary schooling	105 (35.1)	120 (39.9)
Higher education	47 (15.7)	52 (17.3)
**Married or living with a partner**		
Yes	244 (81.6)	244 (81.1)
No	55 (18.4)	57 (18.9)
**Employment status**		
Working outside the household^a^	124 (41.5)	107 (35.5)
Not working outside the household^b^	175 (58.5)	194 (64.5)
**Time since HIV diagnosis**		
< 6 months	93 (31.1)	84 (27.9)
≥ 6 months	206 (68.9)	217 (72.1)
**HIV status disclosure to someone**		
Yes	226 (75.6)	232 (77.1)
No	73 (24.4)	69 (22.9)
**HIV status disclosure to partner**^**c**^		
Yes	172/244 (70.5)	175/244 (71.7)
No	72/244 (29.5)	69/244 (28.3)
**Travel time to clinic**		
< 1 h	214 (71.6)	208 (69.1)
≥ 1 h	85 (28.4)	93 (30.1)
**Clinic of enrolment**		
Chulaimbo	40 (13.4)	40 (13.3)
Huruma	23 (7.7)	25 (8.3)
Kitale	102 (34.1)	103 (34.2)
Matayos	3 (1.0)	2 (0.7)
MTRH	93 (31.1)	93 (30.9)
UGDH	38 (12.7)	38 (12.6)

*MTRH* Moi Teaching and Referral Hospital, *UGDH* Uasin Gishu District Hospital.

^a^Working outside the household i.e. employed, self-employed, casual labour, farm work.

^b^Not working outside the household i.e. unemployed, homemaker, student.

^c^Among participants married or living with a partner (n = 488).

The number of infants tested for HIV by eight weeks of age was 213 in the intervention group (85.5% of the 249 recorded live births in this group) and 216 in the control group (84.7% of the 255 recorded live births in this group). The proportion of infants HIV tested by eight weeks of age out of women enrolled in the trial (intention-to-treat analysis including women who miscarried, had a stillbirth, where lost to follow-up, transferred care, died, or had an infant who died before eight weeks after delivery) was 71.2% in the intervention group and 71.8% in the control group. We did not find a statistically significant difference between participants who received the WelTel intervention and the control group on the uptake of EID HIV testing by eight weeks of age (RR 0.99; 95% CI: 0.90–1.10; *p* = 0.89). Adjustment for age, clinic, and time from HIV diagnosis did not notably change these results (Table 2). In sensitivity analyses excluding participants with evidence of miscarriage, stillbirth, death of the infant or mother, or transfer to a non-study clinic before eight weeks postpartum, similar results were found (Table 2). The number of infants diagnosed with HIV by eight weeks of age was two in the intervention group (0.8% of recorded live births) and three in the control group (1.2% of recorded live births).

**Table 2:** WelTel text message intervention effect on early infant HIV testing.

Outcome	Infant HIV testing 0–8 weeks of age	Infant HIV testing 4–8 weeks of age
WelTel intervention (n = 299)	213 (71.2%)	207 (69.2%)
Routine care (control group, n = 301)	216 (71.8%)	209 (69.4%)
Unadjusted RR (95% CI); *p* value	0.99^a^ (0.90–1.10); *p* = 0.89	1.00^a^ (0.90–1.11); *p* = 0.96
	1.00^b^ (0.92–1.09); *p* = 1.00	1.00^b^ (0.92–1.10); *p* = 0.92
Adjusted RR (95% CI); *p* value	1.00^c^ (0.90–1.10); *p* = 0.95	1.00^c^ (0.90–1.11); *p* = 0.94
	1.00^d^ (0.93–1.09); *p* = 0.94	1.01^d^ (0.92–1.10); *p* = 0.83

*RR* Risk ratio, *CI* Confidence interval.

^a^Unadjusted intention to treat analysis (N = 600).

^b^Cases of stillbirth, miscarriage, death of infant, death of mother and transfer to other facility before eight weeks postpartum excluded (531 participants remained in the analysis).

^c^Adjusted for age, study facility and woman’s time since HIV diagnosis at enrolment (N = 600).

^d^Adjusted for age, study facility and woman’s time since HIV diagnosis at enrolment. Cases of stillbirth, miscarriage, death of infant, death of mother and transfer to other facility before 8 weeks postpartum excluded (531 participants remained in the analysis).

In the additional analysis of infants HIV tested between four to eight weeks of age (intention-to-treat analysis to reduce the impact of at birth HIV testing), 207 infants (69.2% of women enrolled) in the intervention group and 209 infants (69.4% of women enrolled) in the control group were tested (RR 1.00; 95% CI: 0.90–1.11; *p* = 0.96). Similar to the zero to eight week EID testing, the adjusted results did not notably differ (Table 2). In sensitivity analyses excluding participants with evidence of miscarriage, stillbirth, death of the infant or mother, or transfer to a clinic outside the trial before eight weeks postpartum, similar results were found (Table 2). Additionally, we found no evidence to support that trial group allocation was associated with being in the excluded group.

Exploratory hypothesis-generating subgroup analyses investigating effect modification by women’s age, education level, marital and cohabitation status, employment status, time since HIV diagnosis, HIV status disclosure to someone, HIV status disclosure to partner, and time to reach the clinic showed no effect of the WelTel intervention on EID testing and no statistically significant results (Table 3). We found a significant interaction between the WelTel intervention and being enrolled at one of the clinics. In the analysis, the WelTel intervention improved HIV testing by eight weeks of age (RR 1.59; 95% CI: 1.06–2.38; *p* = 0.03), and HIV testing between four to eight weeks of age (RR 1.65; 95% CI: 1.08–2.50; *p* = 0.02) at the UGDH as compared to the MTRH (reference) clinic (Table 3). At the UGDH clinic, 28 infants in the intervention group (73.7% of women enrolled) and 20 infants in the control group (52.6% of woman enrolled) were tested by eight weeks of age.

**Table 3:** Subgroup interaction analyses of WelTel intervention effect on early infant HIV testing (N = 600).

Characteristic of pregnant women at WelTel PMTCT trial enrolment	Subgroup interaction effect on infant HIV testing between 0–8 weeks of age		Subgroup interaction effect on infant HIV testing between 4–8 weeks of age	
	Risk ratio (95% CI)	*p* value	Risk ratio (95% CI)	*p* value
**Age (years)**				
18–24	Ref	Ref	Ref	Ref
25–29	1.01 (0.72–1.42)	0.97	1.04 (0.73–1.49)	0.81
30–34	1.09 (0.79–1.50)	0.60	1.08 (0.77–1.52)	0.66
35–44	1.17 (0.82–1.67)	0.40	1.18 (0.81–1.72)	0.39
**Highest education**				
≤ Primary schooling	Ref	Ref	Ref	Ref
Secondary schooling	0.94 (0.76–1.17)	0.60	0.98 (0.78–1.23)	0.85
Higher education	0.96 (0.71–1.31)	0.80	1.00 (0.72–1.39)	0.99
**Married or living with a partner**				
Yes	1.15 (0.86–1.54)	0.33	1.13 (0.83–1.53)	0.43
No	Ref	Ref	Ref	Ref
**Employment status**				
Working outside the household^a^	1.14 (0.92–1.40)	0.23	1.16 (0.93–1.45)	0.18
Not working outside the household^b^	Ref	Ref	Ref	Ref
**Time since HIV diagnosis**				
< 6 months	1.23 (0.95–1.58)	0.11	1.18 (0.90–1.56)	0.23
≥ 6 months	Ref	Ref	Ref	Ref
**HIV status disclosure to someone**				
Yes	0.93 (0.71–1.22)	0.59	0.98 (0.74–1.31)	0.91
No	Ref	Ref	Ref	Ref
**HIV status disclosure to partner**^**c**^				
Yes	1.12 (0.86–1.45)	0.40	1.15 (0.88–1.52)	0.31
No	Ref	Ref	Ref	Ref
**Travel time to clinic**				
< 1 h	Ref	Ref	Ref	Ref
≥ 1 h	1.00 (0.80–1.25)	0.98	0.97 (0.77–1.23)	0.80
**Clinic of enrolment**				
MTRH	Ref	Ref	Ref	Ref
Chulaimbo + Matayos^d^	1.07 (0.79–1.46)	0.65	1.09 (0.78–1.53)	0.62
Kitale	1.12 (0.87–1.43)	0.39	1.10 (0.85–1.41)	0.47
Huruma	1.23 (0.82–1.86)	0.32	1.05 (0.63–1.77)	0.85
UGDH	1.59 (1.06–2.38)	0.03	1.65 (1.08–2.50)	0.02

*CI* Confidence interval, *MTRH* Moi Teaching and Referral Hospital, *UGDH* Uasin Gishu District Hospital.

^a^Working outside the household i.e. employed, self-employed, casual labour, farm work.

^b^Not working outside the household i.e. unemployed, homemaker, student.

^c^Among participants married or living with a partner (n = 488).

^d^Chulaimbo and Matayos clinics were combined in subgroup analysis for geographic and demographic reasons, due to few participants from the Matayos site.

No adverse events were reported in the intervention group nor the control group at any study site.

## Discussion

In this secondary outcome analysis of EID HIV testing of the WelTel PMTCT trial in western Kenya, we did not find a significant effect of weekly text messages delivered to the mothers on the proportion of infants tested for HIV by eight weeks of age. Moreover, we did not find any significant effect of the WelTel intervention in the exploratory subgroup analyses, except in one clinic that had lower EID uptake in the control group, closer to the EID uptake in Kenya overall^6^. We observed a higher proportion of infants HIV tested by eight weeks of age than the Kenyan national average in 2015 (54%) in both the intervention (85.5%) and control groups (84.7%).

This is the first study evaluating the effect of the WelTel intervention, an interactive text-messaging service, on retention of mother-infant-pairs in PMTCT care. Our results are consistent with two previous RCTs of text message interventions on EID HIV testing rates in PMTCT care conducted in western Kenya including 2472 and 550 pregnant women respectively^21,22^, and a retrospective study of 235 pregnant women from South Africa^23^, that reported no significant effects on EID HIV testing rates in PMTCT care. In contrast to our study, two RCTs of mobile text-messaging conducted before the implementation of WHO’s PMTCT Option B+ in western Kenya^19^ and Mozambique^20^ found modest improvements on EID HIV testing (absolute risk differences of 7% and 6% respectively). The significant improvement in EID HIV testing in an RCT of 388 pregnant women conducted in western Kenya between 2012–2013^19^ could not be reproduced in a larger cluster-RCT of 2472 pregnant women^21^ of a similar intervention in the same study setting conducted five years later. The different study results could be due to policy changes^6^ and overall improvements over time to trace and retain women enrolled in PMTC care in the area, that may be more important for the efficacy of text message interventions than the intervention design or the text message content. Previous RCTs of the WelTel intervention among non-pregnant people living with HIV in Kenya found improvements in ART adherence and HIV viral load suppression^25^, but not in retention in care during the first year of HIV care^26^, the latter outcome being more closely related to early infant HIV testing in PMTCT care.

In our study, we observed higher EID HIV testing rates than national and regional Kenyan testing averages. The EID HIV tests in this trial were collected between 2015 to 2017, and in 2015 the uptake of EID HIV testing in Kenya was 54% by 2 months of age among infants born to women living with HIV^6^. The regional uptakes of routine care EID HIV testing in the counties of the study sites in western Kenya were 41% (Kisumu county), 61% (Uasin Gishu county) and 62% (Busia county) in 2015^6^. The high EID HIV testing uptake in our trial setting is consistent with the two cluster-RCTs from western Kenya^21,22^, possibly indicating a positive overall impact on introducing mobile phone communication and text-messaging in the study sites, as suggested in an RCT from 2018 of a text-messaging intervention of 388 women in western Kenya, where the EID testing rate was significantly higher in the routine care group compared to 727 women who were not enrolled in that trial^35^. Participation in a clinical trial may generate increased attention and motivation as all women underwent interview procedures at trial enrolment. In our trial, we had higher enrolment than expected (82%), which could indicate that women in general were highly motivated to participate in PMTCT care resulting in high EID HIV testing rates in both groups.

Other possible reasons are lower barriers to testing due improved partner disclosure of HIV status and diminished concerns of intimate partner violence^36^. We have previously observed that pregnant women in this study setting are more willing to disclose their HIV status to a current partner^37^ compared to earlier studies^38–44^, which is consistent with other recently published studies from Kenya^45–47^, indicating a potential positive move forward towards more openness between partners. We have also reported on reduced concerns of intimate partner violence related to women’s participation in PMTCT care in the study area^37^. Moreover, the proportion of infants diagnosed with HIV by eight weeks of age in our trial was only 1.0%, which is much lower than the national EID HIV transmission rate (5%) by eight weeks of age among HIV-exposed infants in 2019^1^. This could also be an indication of reductions of barriers to testing with increasing disclosure and openness about HIV to partners^37^, reduction in stigma among women in PMTCT care^48^, and program improvements resulting in improved ART adherence among women enrolled in PMTCT care^1,6^. The different transmission rates (in Kenya vs. in our trial) are however not fully comparable since 6% of pregnant women living with HIV in Kenya did not receive ART in 2019^1^, a subpopulation that may have contributed disproportionally to the the MTCT of HIV.

Despite being much higher than the national average, an 85% uptake of EID testing indicates that further improvement is needed to reach the UNAIDS’ goal of eliminating MTCT^49^. The relatively high uptake of EID HIV testing in our study is encouraging, but may have decreased our ability to detect an effect of the WelTel intervention. This may be why we found a statistically significant result at the UGDH clinic, where the EID HIV testing in the control group was much lower compared to testing in the control group as a whole. The UGDH clinic is a county hospital serving rural and urban areas. There were no differences in PMTCT services compared to the other clinics during the trial period. Since there were no significant differences in EID HIV testing rates between the intervention and control group at other ANC clinics or in other subpopulations, the significant result may also be a type I error.

Our results also needs to be interpreted in the context of policy changes made during the trial period as it may have impacted the study findings and improved overall retention in care and uptake of EID HIV testing. Before and during the trial, several healthcare interventions were implemented at the ANC clinics involved in the study to improve the quality, the uptake of PMTCT care, and retention in PMTCT programs. A defaulter tracing outreach program began in 2005 and has become part of routine PMTCT care, where women who default on their appointments are traced (first by telephone, then by household visit) and encouraged to return to care^30,31^. A mentor mother program was also initiated in 2013 for counselling and peer-support of pregnant women living with HIV^6^. During the trial, beginning in 2015, PMTCT care was integrated with regular ANC care with the aim of decreasing HIV related stigma due to visiting HIV specific clinics^6,50^. Another study from Kenya observed improvements in the uptake of EID HIV testing after implementation of a mentor mother and defaulter tracing program to improve PMTCT care^21^. An RCT of 372 women in PMTCT care in Nigeria also found that implementation of a package of similar changes, including integration of care, led to significant improvements in PMTCT outcomes including six- and 12-week postpartum retention in care^51^.

Unpublished focus group evaluations with healthcare workers that we conducted at the WelTel PMTCT sites revealed an overall increased use of mobile phone communication during the trial period, as part of routine care for trial- and non-trial participants. This included text-messaging patients about laboratory results, phone calls as reminders of upcoming clinic appointments, as well as healthcare workers providing patients with their personal contact information so that they could call them about health-related issues at any time. These reports of high and increasing overall telecommunication between participants in the control group and clinic staff during the trial are consistent with the 2015 Kenyan guidelines for ART providers to increase their use of mobile phones to communicate with patients^31^. This may have impacted our results, possibly undermining our chances to detect an effect of the intervention on EID HIV testing. Substantial increases in direct communication between participants in the control group and community health workers were also observed in two previous RCTs of text message interventions in Kenya^22,25^.

A key strength of this study is its high participation rate, and multicentre design including both urban and rural centres across a large area in western Kenya, contributing to the generalizability of the study results. The intervention was implemented to closely resemble real-life conditions. No incentives were given to the participants in the trial and all contact with the participants related to the text message intervention was handled by clinic staff. The study also has several limitations. We were not able to quantitatively measure non-intervention telecommunication between clinic staff and participants; therefore, we are unable to assess the extent to which text-messaging between staff and participants increased in the control group. Despite no reports of it, components of the intervention may have been implemented in the control group due to the open-label design and the handling of all telecommunication with the participants by staff at the clinics. An increased overall use of telecommunication between patients and healthcare staff, and a defaulter tracing program for women who missed PMTCT care appointments may have attenuated any effect that the text-messaging intervention may have had on EID HIV testing rates. In the current study, we did not assess if the intervention or control group differed in terms of outreach follow-up needed to ensure that EID HIV testing was done. We used sealed envelopes for randomisation due to logistical reasons in this study setting. There were no reports of pre-opened envelopes or unconcealed randomisation sequence due to fixed block sizes, and a good balance of baseline characteristics between the groups (Table 1) further supports that the randomisation was successful.

Further studies may be warranted to investigate the effect of interactive text message interventions to increase EID HIV testing in settings with higher barriers to testing (including those with higher rates of non-disclosure of HIV status to a partner) and in settings without defaulter tracing or other support mechanisms to promote retention in PMTCT care. An important outcome, that we were unable to measure in this study, was if proactive automated interactive text-messaging was more efficient than tracing patients after they default on clinic visits. Given higher use of mobile phones for healthcare in general, future research may focus more on potential programmatic and cost efficiencies of structured versus unstructured digital health messaging services. Other quality improvement and implementation outcomes in the study protocol will be reported separately.

## Conclusion

We found no evidence to support that the WelTel intervention improved EID HIV testing. A higher uptake of EID testing than expected in both groups may be a result of lower barriers to EID testing and improved PMTCT care in western Kenya, including the broader standard use of mobile phone communication between healthcare workers and patients.

## Data Availability

The datasets used and/or analysed during the current study are available from the corresponding author on reasonable request.

## Abbreviations

AMPATH: Academic Model Providing Access to Healthcare
ANC: Antenatal care
ART: Antiretroviral therapy
CI: Confidence interval
EDCTP: European-Developing Countries Clinical Trial Partnership
EID: Early infant diagnosis
ISRCTN: International Standard Randomised Controlled Trial Number
MTCT: Mother-to-child transmission
MTRH: Moi Teaching and Referral Hospital
NASCOP: Kenyan National AIDS and STI Control Program
PMTCT: Prevention of mother-to-child transmission
RCT: Randomised controlled trial
RR: Risk ratio
SSA: Sub-Saharan Africa
UGDH: Uasin Gishu District Hospital
UNAIDS: Joint United Nations Programme on HIV/AIDS
WHO: World Health Organization
